# An Instructive Case of Hypertrophied Pulp

**Published:** 1904-09

**Authors:** Leonard C. Hales

**Affiliations:** Wellington, New Zealand


### AN INSTRUCTIVE CASE OF HYPERTROPHIED PULP.

BY LEONARD C. HALES, D.D.S., WELLINGTON, NEW ZEALAND.

Miss B. became a patient of mine in December, 1903. After making a careful examination of the teeth I noted that although most of the anterior teeth were free from caries, the molars, both first and second, on both the upper and lower jaws, and on either side of the mouth, were badly decayed.

The patient, being about twelve years of age, naturally had her permanent teeth in position. After a thorough cleansing of the organs of mastication, I took the lower left first permanent molar, which was badly decayed and seemed to be giving the most trouble, the cavity of decay taking in the greater part of the occlusal surface and the mesial wall, along with the lingual wall, on which side the cavity extended well under the gum margin.

The shell that remained was filled with a large bulbar mass of pulp-tissue, and which I recognized at once to be a far-advanced case of hypertrophied pulp.

Contrary to my expectations, I found the tissue to be abnormally sensitive, and suffused with blood, which seemed to gush out when the part was touched. I tried cocaine in the dry crystalline form, but it had no effect, as the pulp did not seem to absorb it. I then made an application of trichloracetic acid, but its action, apart from being exceedingly slow, gave the patient intense pain, so I removed the agent; and as I noticed that the patient was fast arriving at an unpleasant nervous condition, I at once resorted to Dr. Maercklen's suggestion, and, packing the parts with iodine crystals, I made a cap of base-plate gutta-percha, closed the cavity, and told the patient to return in two days' time.

Two days later the patient returned; the pulp was less sensitive, but still bled profusely. I was enabled, however, to remove the larger portion of it with a sharp excavator, and, making another application of the iodine crystals, I closed the cavity and dismissed the patient for two days.

When the patient came again I was enabled, with the aid of a hypodermic syringe, to inject the root-canals and pulp-chamber with a cocaine solution with comparatively little pain to the patient. I then extirpated the remainder of the pulp-tissue, thoroughly cleansed the canals, inserted a dressing of oil of eucalyptus in the canals, and again dismissed the patient, this time for a period of four days.

When the patient returned I removed the dressing and filled the canals with oxychloride of zinc, and after allowing the tooth to remain stopped with gutta-percha for several weeks, with no signs of trouble, I inserted an amalgam filling. The tooth has remained comfortable ever since, and to all appearances will continue to do so.

